# A meta-analysis of the risk of venous thromboembolism in inflammatory rheumatic diseases

**DOI:** 10.1186/s13075-014-0435-y

**Published:** 2014-09-25

**Authors:** Jason J Lee, Janet E Pope

**Affiliations:** Schulich School of Medicine, Western University, St Joseph’s Health Care, 268 Grosvenor Street, London, ON N6A 4V2 Canada; Division of Rheumatology, St Joseph’s Health Care, 268 Grosvenor Street, London, ON N6A 4V2 Canada

## Abstract

**Introduction:**

We performed a meta-analysis to investigate the risk of deep vein thrombosis (DVT) and/or pulmonary embolisms (PEs) in patients with inflammatory arthritis, vasculitis and connective tissue diseases (CTDs) (systemic lupus erythematosus (SLE), Sjögren’s syndrome, inflammatory myositis and systemic sclerosis (SSc)).

**Methods:**

PubMed, Embase, the Cochrane databases and MEDLINE were searched to identify full-text English-language publications about adult patients with rheumatologic inflammatory diseases and venous thromboembolisms (VTEs). Data regarding rates of DVTs and PEs were extracted. Using random-effects models, pooled estimates for VTEs in individual and pooled diseases were compared with matched populations where possible. Studies were excluded if VTEs were described in the setting of pregnancy, postoperative outcomes or solely antiphospholipid antibody syndrome.

**Results:**

Most of the 5,206 studies were excluded because they did not state the rate or incidence of VTEs. In total, 25 studies remained for analysis. Ten studies that included rheumatoid arthritis comprised an aggregate of 5,273,942 patients and 891,530,181 controls with a cumulative VTE incidence of 2.18% (95% confidence interval (CI): 1.82% to 2.54%) and an odds ratio of 2.23 (95% CI: 2.02 to 2.47) compared to age- and sex-matched populations. Ten studies comprised an aggregate of 54,697 SLE patients with a cumulative VTE incidence of 7.29% (95% CI: 5.82% to 8.75%). Four Sjögren’s syndrome studies comprising an aggregate of 25,100 patients demonstrated a cumulative VTE incidence of 2.18% (95% CI: 0.79% to 3.57%). Four studies of inflammatory myositis comprising an aggregate of 8,245 patients yielded a cumulative VTE incidence of 4.03% (95% CI: 2.38% to 5.67%). The SSc- and antineutrophil cytoplasmic antibody vasculitis–related cumulative VTE rates (four studies each) were 3.13% and 7.97%, respectively.

**Conclusions:**

The inflammatory rheumatologic diseases studied were all associated with high rates of VTEs—more than three times higher than in the general population.

**Electronic supplementary material:**

The online version of this article (doi:10.1186/s13075-014-0435-y) contains supplementary material, which is available to authorized users.

## Introduction

Venous thromboembolism (VTE) is a vascular phenomenon that includes clinical entities such as deep vein thrombosis (DVT) and pulmonary embolism (PE). These venous coagulopathies usually occur in the setting of Virchow’s triad, which describes conditions in which thromboses develop as a consequence of stasis, endothelial injury and innate hypercoagulability [1,2]. It is increasingly becoming recognized that active inflammation is an important process that increases coagulability and leads to thrombosis [3]. Active inflammation is a prothrombotic state characterized by upregulation of tumor necrosis factor α (TNF-α) and activation of endothelial cells. It is thought that upregulation of TNF-α increases tissue factor in the serum, which is a natural procoagulant, while downregulating protein C, which is a natural anticoagulant [3]. Also, activation of endothelial cells promotes platelet activation, which is important for thrombus formation.

Rheumatologic conditions are often inflammatory by nature. However, despite this mechanistic link between rheumatologic diseases and VTEs, these highly inflammatory conditions may be under-recognized as risk factors for hypercoagulability. The only exception is the well-known association between antiphospholipid antibodies in systemic lupus erythematosus (SLE) and both venous and arterial thromboses. There are several case reports, retrospective cohort studies and prospective observational analyses highlighting the increased risk of VTE in patients with rheumatologic diseases [4-9]. Most of the data in the literature reveal this concern in patients with rheumatoid arthritis (RA) and SLE, and the SLE studies are focused mostly on increased risks associated with positive antiphospholipid status rather than on the innate hypercoagulability nature of this inflammatory disease [8,10]. VTEs seem to be linked to disease activity and/or inflammation in many of the inflammatory rheumatologic diseases.

Patients who develop VTEs have high rates of morbidity and mortality [2]. The incidence of first-time VTE in the United States is about 1 in 1,000 person-years [1]. Therefore, it is important to understand the excess magnitude of this issue in patients with inflammatory rheumatologic diseases. Ideally, the modifiable risk factors would be known and altered and poor outcomes mitigated. To investigate these issues, we conducted a meta-analysis of the risk of developing DVT and/or PE in patients with inflammatory arthritis, vasculitis and connective tissue diseases (CTDs) such as SLE, Sjögren’s syndrome, inflammatory myositis and systemic sclerosis.

## Methods

### Search strategy

We performed a literature search of English-language publications related to VTE, DVT and/or PE in patients with inflammatory arthritis, vasculitis and CTDs such as SLE, Sjögren’s syndrome, inflammatory myositis and systemic sclerosis. We searched for articles in MEDLINE, Embase, PubMed and the Cochrane databases from their inception (1966, 1950, 1980 and 1991, respectively) to June 2014 (Additional file 1). All studies that included thrombosis in the setting of inflammatory rheumatologic diseases were collected. Inclusion criteria included articles published in English with rates of VTE in adult patients with the aforementioned rheumatologic diseases. Exclusion criteria included articles reporting VTE in the setting of antiphospholipid antibodies, pregnancy-related outcomes and postoperative outcomes. The details of our search strategy are given in Additional file 1.

### Description of studies

Both authors agreed on which studies were included and excluded. Data from each study were extracted by one investigator (JJL). The following information was systematically extracted: first author, year of publication, country where the study was done, total number of patients included (cases and controls), total number of VTE events observed in the inflammatory rheumatologic disease, total number of control patient population where available and total number of VTE events observed in the control cohort where available. Some studies included multiple patient populations with various rheumatologic diseases. Therefore, from those articles, data were extracted for each disease and analyzed separately.

### Quality assessment

Study quality was assessed by using the Strengthening the Reporting of Observational Studies in Epidemiology (STROBE) checklist for cohort, case–control and cross-sectional studies. The STROBE checklist consists of 22 items; 18 items are common to all three study designs, and 4 items (6a/6b, 12d, 14c and 15) are specific to cohort, case–control and cross-sectional studies. The maximum score varies based on the number of applicable items on the checklist. Two items on the checklist (items 12e and 16c) pertain to statistical methods. Item 12e describes any sensitivity analysis of the main results, and, for item 16c, where relevant, the relative risk is translated into absolute risk for a meaningful time period. Therefore, these items were not relevant to all of the articles. One item applied only to matched studies (item 6b) and another only to cohort studies (item 14c). The maximum attainable score is 32. The purpose of STROBE is not to give a quality score, but to ensure clear presentation of reporting.

### Statistical analysis

Forest plots of VTE rates were graphed separately from the available articles. Estimates of VTEs by sample size were examined to determine if larger samples had fewer outlying estimates. Confidence intervals (CIs) at the 95% level for study estimates were calculated using Wilson’s scoring method [11]. Within-study variance was estimated by using the formula Vari = *p*_i_*(1 − *p*_i_)/*N*_i_ [12], where *p*_i_ is the estimate of the true proportion of patients with the condition for study i and *N*_i_ is the total sample size of study i [12]. Study weights were calculated as the inverse of the within-study variance. A random-effects meta-analysis was used to pool study proportion estimates while accounting for differences in study quality, study population and study design [13]. The *I*^2^ statistic was used to quantify the magnitude of between-study heterogeneity [14]. An *I*^2^ value represents the percentage of total variation across studies due to true difference rather than to chance, with values of 0% to 30%, 31% to 50%, and >50% representing mild, moderate and notable heterogeneity, respectively [14]. The τ^2^ value was the square root of the between-study variance, and the *P*-value was calculated with Cochrane’s Q test, the classic measure of heterogeneity. Statistical analyses were performed using R version 2.0.1 (R Foundation for Statistical Computing, Vienna, Austria). Many of the studies included in the meta-analysis did not report a control group; therefore, relative risks and odds ratios could not be calculated for the diseases described in those articles.

## Results

### Search results

**Figure 1:**
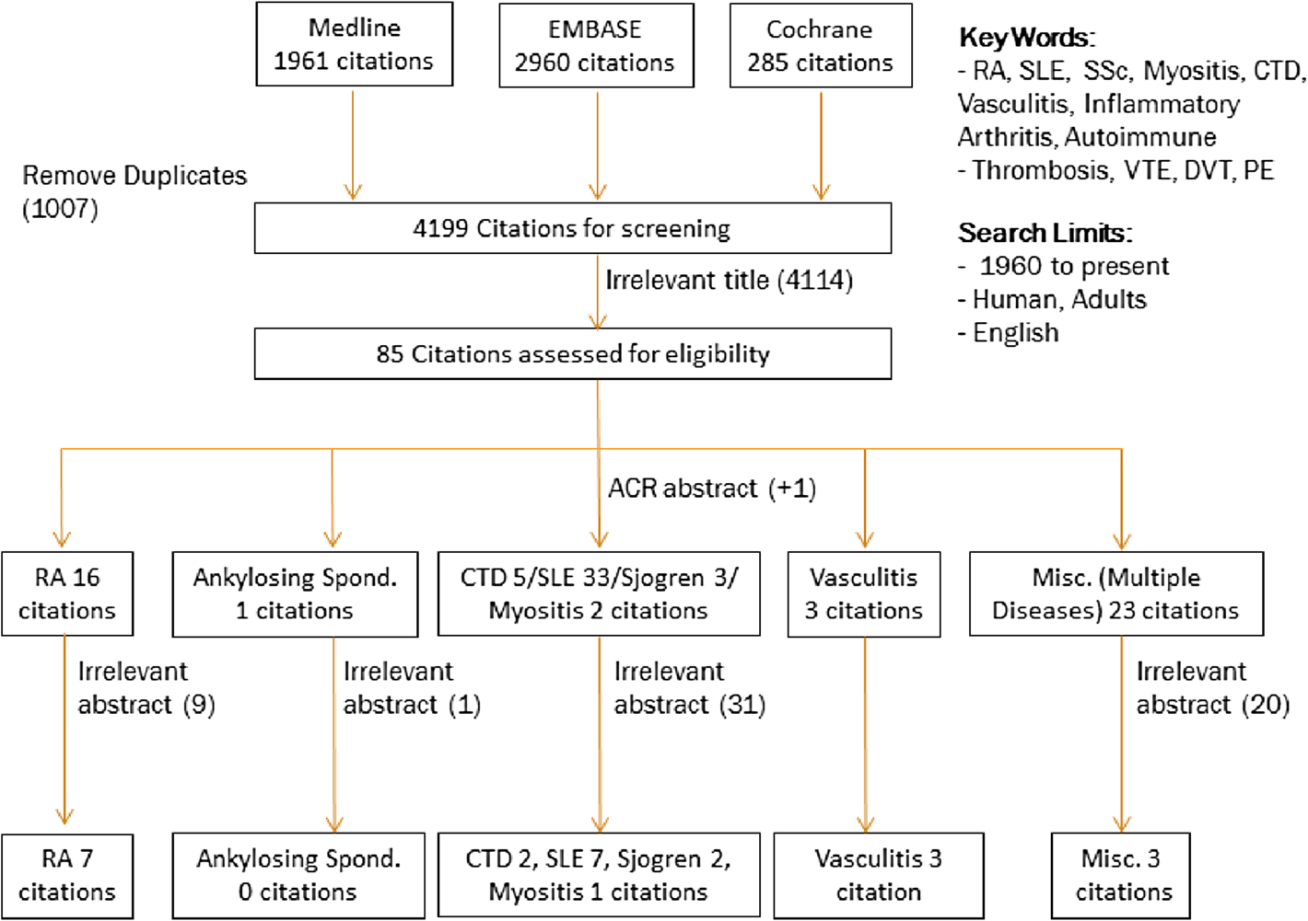
**Flowchart of the search strategy, selection process and number of articles identified, excluded and included for analysis.** ACR, American College of Rheumatology; CTD, Connective tissue disease; DVT, Deep vein thrombosis; PE, Pulmonary embolism; RA, Rheumatoid arthritis; SSc, Systemic sclerosis; SLE, Systemic lupus erythematosus; VTE, Venous thromboembolism.

In the search process, we identified 5,206 articles (Figure 1). During the title review process, 1,007 articles were removed as duplicate citations from different databases. Next, during the abstract review process, 4,114 articles were eliminated because they were irrelevant (that is, not related to VTE). Subsequently, one new article, pending publication and based on an abstract presented at the 2013 American College of Rheumatology meeting [15], was included. The exclusion criteria were applied to the remaining 85 unique articles, and 60 studies were excluded. Thus, the final review included 25 unique articles [4-9,15-33] (Table 1).

**Table 1:** **Characteristics of studies included with descriptive statistics on rates of venous thromboembolism** ^**a**^

**Study**	**Year**	**Location/source**	**Country**	**Total RA patients**	**RA patients with VTE**	**Total controls**	**Controls with DVT**
Rheumatoid arthritis							
Choi *et al*. [5]	2013	The Health Improvement Network	UK	9,589	192	95,776	870
Bacani *et al*. [6]	2012	Olmsted County, Minnesota	USA	464	19	464	7
Holmqvist *et al*. [7]	2012	National Patient Register	Sweden	39,372	838	173,417	1,866
Matta *et al*. [16]	2009	National Hospital Discharge Survey	USA	4,818,000	110,000	891,055,000	10,227,000
Liang *et al*. [17]	2006	Olmsted County, Minnesota	USA	609	38	N/A	N/A
Chung *et al*. [18]	2013	Taiwan National Health Insurance Research Database	Taiwan	29,238	278	116,952	394
Kim *et al*. [19]	2013	US insurance claims	USA	22,143	265	88,572	448
Romero-Díaz *et al*. [20]	2009	Instituto Nacional de Ciencias Médicas y Nutrición Salvador Zubirán	Mexico	156	3	N/A	N/A
Zöller *et al*. [21]	2012	MigMed2 database	Sweden	86,366	2,500	N/A	N/A
Ramagopalan *et al*. [22]	2011	Oxford Record Linkage Study	UK	268,005	6,825	N/A	N/A
Systemic lupus erythematosus							
Aviña-Zubieta *et al*. [4]	2012	University of British Columbia	Canada	5,031	265	50,310	533
Calvo-Alén *et al*. [8]	2005	LUMINA Study Group	USA	570	51	N/A	N/A
Kaiser *et al*. [9]	2009	UCSF Lupus Genetics Project	USA	1,930	426	N/A	N/A
Romero-Díaz *et al*. [20]	2009	Instituto Nacional de Ciencias Médicas y Nutrición Salvador Zubirán	Mexico	241	25	N/A	N/A
Zöller *et al*. [21]	2012	MigMed2 database	Sweden	9,147	276	N/A	N/A
Ramagopalan *et al*. [22]	2011	Oxford Record Linkage Study	UK	23,544	636	N/A	N/A
Sarabi *et al*. [23]	2005	University of Toronto Lupus Database	Canada	544	30	N/A	N/A
Chang *et al*. [24]	2006	McGill University Health Centre Lupus Clinic Registry	Canada	426	40	N/A	N/A
Gladman and Urowitz [25]	1980	Lupus Clinic at the Wellesley Hospital	Canada	180	17	N/A	N/A
Chung *et al*. [32]	2014	Taiwan National Health Insurance Research Database	Taiwan	13,084	228	52,336	61
Sjögren’s syndrome							
Zöller *et al*. [21]	2012	MigMed2 database	Sweden	3,410	100	N/A	N/A
Ramagopalan *et al*. [22]	2011	Oxford Record Linkage Study	UK	12,680	305	N/A	N/A
Haga *et al*. [26]	2008	Haukeland University Hospital	Norway	90	4	N/A	N/A
Chung *et al*. [31]	2014	Taiwan National Health Insurance Research Database	Taiwan	8,920	59	35,680	98
Inflammatory myositis							
Romero-Díaz *et al*. [20]	2009	Instituto Nacional de Ciencias Médicas y Nutrición Salvador Zubirán	Mexico	24	2	N/A	N/A
Zöller *et al*. [21]	2012	MigMed2 database	Sweden	2,122	92	N/A	N/A
Ramagopalan *et al*. [22]	2011	Oxford Record Linkage Study	UK	6,002	167	N/A	N/A
Selva-O’Callaghan *et al*. [27]	2011	Vall d’Hebron General Hospital	Spain	97	9	N/A	N/A
Systemic sclerosis							
Zöller *et al*. [21]	2012	MigMed2 database	Sweden	9,323	370	N/A	N/A
Ramagopalan *et al*. [22]	2011	Oxford Record Linkage Study	UK	11,643	244	N/A	N/A
Aviña-Zubieta *et al*. [15]	2013	University of British Columbia	Canada	1,284	73	12840	197
Chung *et al*. [33]	2014	Taiwan National Health Insurance Research Database	Taiwan	1,895	22	7,580	12
ANCA-associated vasculitis							
Zöller *et al*. [21]	2012	MigMed2 database	Sweden	15,085	878	N/A	N/A
Stassen *et al*. [28]	2008	University of Groningen	Netherlands	198	23	N/A	N/A
Merkel *et al*. [29]	2005	The Wegener’s Granulomatosis Etanercept Trial Research Group	USA	167	16	N/A	N/A
Allenbach *et al*. [30]	2009	French Vasculitis Study Group Cohort	France	845	67	N/A	N/A

^a^ANCA, Antineutrophil cytoplasmic antibody; DVT, Deep vein thrombosis; N/A, Not available; RA, Rheumatoid arthritis; VTE, Venous thromboembolism.

### Studies included

Ultimately, 25 articles were subject to full review. Of the 25 articles, seven were from the United States [6,8,16,17,19,29], five were from Canada [4,15,23-25], four were from Taiwan [18,31-33], two were from the United Kingdom [5,22], two were from Sweden [7,21], one was from Mexico [20], one was from Spain [27], one was from Denmark [26], one was from the Netherlands [28] and one was from France [30]. Several studies included multiple rheumatologic diseases [15-17], and data were extracted according to disease. The included articles are summarized in Table 1 according to disease, along with descriptive statistics on the cumulative incidence of VTEs. The maximum attainable score was 32 for the STROBE checklist, and the mean score for 24 articles was 26.5 (range: 24 to 30), not including the one abstract from the 2013 American College of Rheumatology meeting [15].

### Analyses

**Figure 2:**
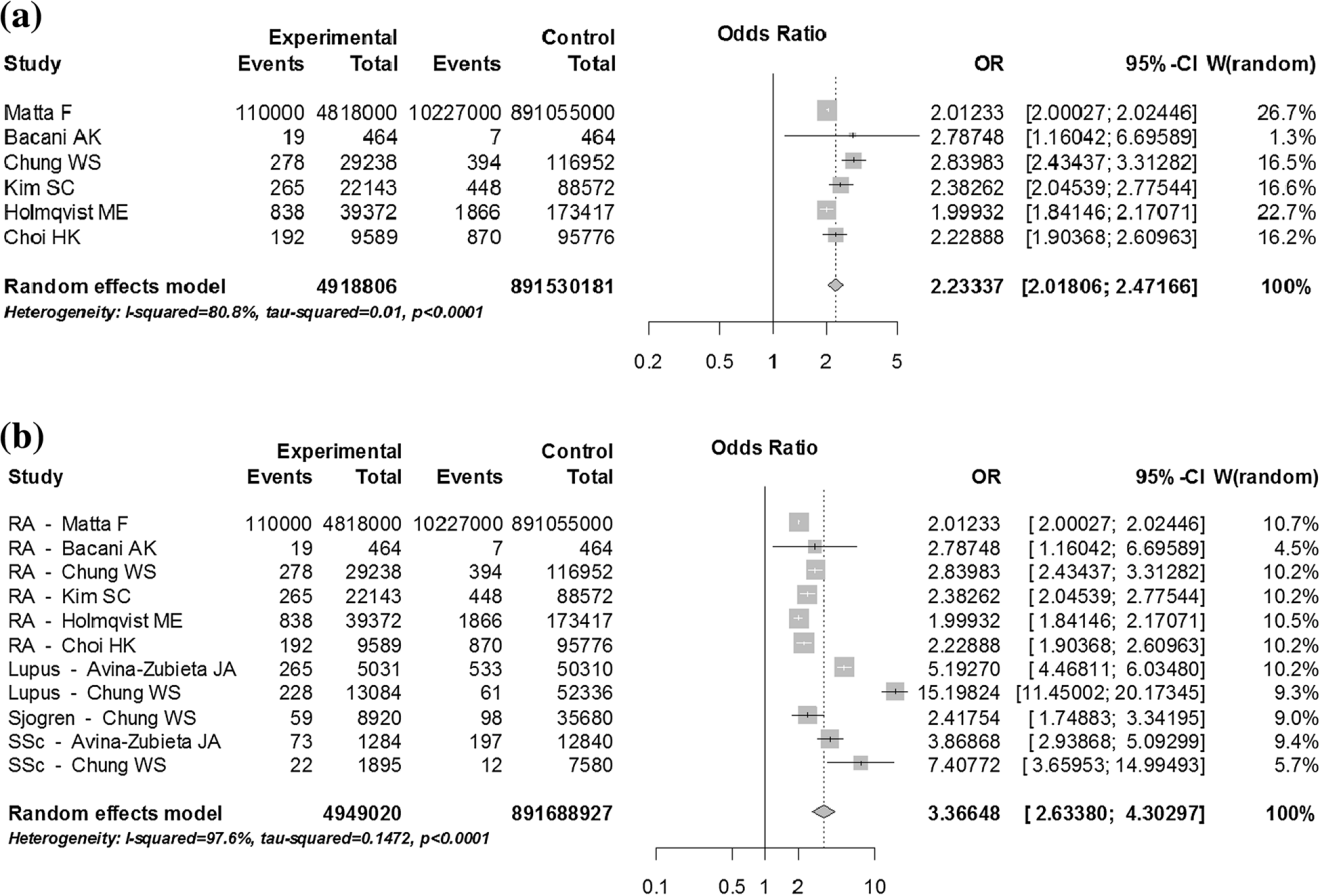
**Forrest Plots of VTE rates and odds ratios in patients with rheumatologic diseases.** **(a)** Cumulative Incidence of VTEs in RA compared to matched control population presented as Odds Ratio. **(b)** Cumulative Incidence of VTEs in patients with RA, SLE, Scleroderma compared to matched control population presented as Odds Ratio.

Of the 25 studies, 10 included patients with RA. In the aggregate, these studies identified a total of 5,273,942 patients and 891,530,181 controls with a VTE cumulative incidence of 2.18% (95% CI: 1.82% to 2.54%). The odds ratio of RA patients’ developing DVT and/or PE was 2.23 (95% CI: 2.02 to 2.47) compared to age-, sex- and other comorbidity-matched populations such as diabetes mellitus, peripheral vascular disease and/or coronary artery disease, and malignancy. In 10 studies that included patients with SLE, researchers observed an aggregate of 54,697 patients with a VTE cumulative incidence of 7.29% (95% CI: 5.82% to 8.75%). Four Sjögren’s syndrome studies with an aggregate of 25,100 subjects demonstrated a VTE cumulative incidence of 2.18% (95% CI: 0.79% to 3.57%). Four studies of inflammatory myositis (dermatomyositis and/or polymyositis) (*N* =8,245) resulted in a VTE cumulative incidence of 4.03% (95% CI: 2.38% to 5.67%). Four studies of antineutrophil cytoplasmic antibody (ANCA)–associated vasculitis (*N* =16,295) resulted in a VTE cumulative incidence of 7.97% (95% CI: 5.67% to 10.28%). Four studies analyzed VTE rates for systemic sclerosis (*N* =24,145), which resulted in a VTE cumulative incidence of 3.13% (95% CI: 1.73% to 4.52%). There were no independent data available for other inflammatory arthritides such as spondyloarthropathies. Overall, all inflammatory rheumatologic diseases were associated with high rates of VTEs (Figure 2).

## Discussion

Patients with inflammatory rheumatologic diseases are at increased risk of developing VTEs. To our knowledge, our present meta-analysis is the first to show this increased inherent risk in patients across all inflammatory rheumatologic diseases. This increased risk was seen in studies of hospitalized and nonhospitalized patients. Specifically, patients with RA, SLE, inflammatory myositis, Sjögren’s syndrome, ANCA-associated vasculitis and systemic sclerosis appear to have this increased risk. There was insufficient data in the literature for us to comment on risks for other inflammatory conditions.

Certainly, patients with various rheumatologic illnesses may be at increased risk of developing VTEs for several different reasons. Patients with inflammatory joint diseases are more likely to be immobile because of pain, especially during times of active joint inflammation. Also, these patients are more likely to undergo surgeries such as joint arthroplasty procedures. We attempted to account for these confounders in this meta-analysis by excluding VTE rates reported in the perioperative setting. The inherent hypercoagulable nature of inflammatory autoimmune diseases is often overlooked [1,2]. This is despite recent evidence which suggests that active inflammation is a strong driving force that leads to local and systemic imbalances in the coagulation system [3].

The data presented in our present study are clinically significant when considering the morbidity and mortality associated with VTEs [1,2]. In a recently published epidemiological study by Tagalakis *et al*. [2], the 1-year survival rate of unprovoked VTEs was estimated to be only 93%. For patients who developed a VTE secondary to a major risk factor such as recent surgery or hospitalization, the 1-year survival rate was only 84%. If the patient survives a VTE episode, the subsequent cost of rehabilitation and long-term anticoagulation is great. Also, by recognizing active inflammation as a risk factor for VTEs, the duration of anticoagulation may be adjusted once an event has occurred. This may be an area of future research in both rheumatology and hematology.

Recognizing a risk factor is only part of the battle. The natural follow-up to the results reported here is to determine factors important in prevention and when, if at all, it is appropriate to use prophylaxis. There are limited and scant data in the literature regarding this issue. Most of the literature regarding prophylaxis against VTEs in rheumatology patients focuses on antiphospholipid antibody status. Specifically, hydroxychloroquine has shown promise in reducing the incidence of VTEs in patients with SLE [34,35]. Aspirin also seems to be beneficial in the setting of antiphospholipid antibodies, particularly during pregnancy [36]. However, there are no studies in the current literature in which the benefit of aspirin in rheumatologic patients without antiphospholipids in the serum has been assessed.

We believe that the increased VTE risk is associated with the activity of the inflammatory diseases rather than with the treatments used for controlling the disease. There are two major reasons for this hypothesis. First, some of these studies show that the risk of VTE is highest in the first year of disease, with a significant drop-off in subsequent years. Some postulate that this is because the disease is most active in the first year, with improvement occurring once inflammation is controlled by medications. Second, studies of vasculitis, such as ANCA-associated vasculitides and giant cell arteritis show that the risk of thrombosis is lower when the disease is treated with glucocorticoids and disease-modifying agents. Therefore, we believe that the risk of thrombosis is associated with the disease. However, further studies are required to confirm this hypothesis.

To our knowledge, this meta-analysis is the first comprehensive study to recognize the hypercoagulability risk in patients with many inflammatory rheumatologic conditions. Notable limitations of this meta-analysis include possible publication bias. Due to underrecognition of this issue in the published literature and in practice, negative studies may not have been available for inclusion. However, under the same principle, rates of VTE in the published literature may underestimate true rates. Clinical signs of VTEs, especially DVTs, are often subtle. Patient-reported symptoms may be vague and may even be misattributed to their rheumatologic disease. Another potential limitation of this meta-analysis involves lack of prospective data that include disease activity, other comorbidities and confounders. Particularly in studies without control comparators, it may be difficult to isolate patients’ rheumatologic diseases as the sole cause for VTEs.

With regard to the increased risk in patients with SLE- and ANCA-associated vasculitis, we also recognize that their risk is substantially higher compared to the other disease populations. For ANCA-associated vasculitides, we felt that the risk was attributable to a combination of (1) direct vessel injury from inflammation (vasculitis), (2) greater local edema and vascular narrowing secondary to vascular inflammation and remodeling and (3) overall inflammatory state, as in all other inflammatory diseases. Of course, further investigations are required to prove this theory. With regard to the increased risk in SLE, again it is likely a multifactorial issue, including renal involvement (such as nephrotic syndrome, which can increase hypercoagulability by an imbalance in excreting antithrombotic factors), an increased prevalence of antiphospholipid antibodies and an overall inflammatory state, as in all other inflammatory diseases leading to endothelial dysfunction and via other mechanisms. Specifically with regard to antiphospholipid antibody status, we tried to mitigate this confounder as much as possible by excluding studies that directly tested for hypercoagulability in the setting of known antiphospholipids in the serum. However, we recognize that the studies included did not specifically test for or exclude antiphospholipid status, so it is probable that at least some of the increased VTE risk in SLE patients is due to antiphospholipid antibodies. The prevalence of antiphospholipid syndrome in SLE patients can vary by cohort, so part of the usual lupus cohort studied would have one-fourth with antiphospholipid antibodies and some with renal lupus. In general, none of the included studies reported disease activity in relation to the VTE events, so we were unable to perform subanalyses in this regard.

In our present meta-analysis, we pooled the studies using a random-effects model. However, high heterogeneity was still observed, owing to the wide differences in patient populations in each cohort. These differences included ethnicity, age, duration of disease, activity of disease, setting of VTE (that is, inpatient vs. outpatient), environmental factors and other unknown variables. We tried to minimize the variance by including large studies with clearly defined patient characteristics. Still, there were a limited number of studies from which data were pooled. However, for all inflammatory rheumatic diseases for which data were extracted, the rate of VTEs was higher than expected, with a pooled increased odds ratio of 3.4.

## Conclusion

In this study, we quantified the elevated risk of venous thromboembolic events in patients across the included inflammatory rheumatologic diseases. Although we provide strong evidence for this elevated baseline risk in the rheumatology patient population, identifying high-risk patients within each disease and reducing the risk by treatment of disease activity or antiplatelet prophylaxis cannot be determined on the basis of our meta-analysis.

## Additional file

Additional file 1:
**Full literature search strategies and full description of the database search strategies.**

## Abbreviations

ANCA: Antineutrophil cytoplasmic antibody
CTD: Connective tissue disease
DVT: Deep vein thrombosis
PE: Pulmonary embolism
RA: Rheumatoid arthritis
SLE: Systemic lupus erythematosus
SSc: Systemic sclerosis
STROBE: Strengthening the Reporting of Observational Studies in Epidemiology
TNF-α: Tumor necrosis factor α
VTE: Venous thromboembolism
