# Supplementary material for: A meta-analysis of the risk of venous thromboembolism in inflammatory rheumatic diseases
Source: Arthritis Res Ther. 2014 Sep 25;16(5):435. doi: 10.1186/s13075-014-0435-y (PMC4207310; doi:10.1186/s13075-014-0435-y)
Supplement: Additional file 1: — Full literature search strategies and full description of the database search strategies. [file 13075_2014_435_MOESM1_ESM.docx]

Additional File 1: Full Search Strategies

**Cochrane Final Strategy**

Database: EBM Reviews - Cochrane Central Register of Controlled Trials <June 2014>, EBM Reviews - Cochrane Database of Systematic Reviews <2005 to June 2014>, EBM Reviews - Cochrane Methodology Register <3rd Quarter 2012>, EBM Reviews - Database of Abstracts of Reviews of Effects <2nd Quarter 2014>, EBM Reviews - Health Technology Assessment <2nd Quarter 2014>, EBM Reviews - NHS Economic Evaluation Database <2nd Quarter 2014>

Search Strategy:

--------------------------------------------------------------------------------

1 Thrombosis/ (1182)

2 Thromboembolism/ (902)

3 Venous Thromboembolism/ (259)

4 exp Venous Thrombosis/ (2052)

5 exp Pulmonary Embolism/ (756)

6 (thrombo* or thrombus* or embol*).tw. (22487)

7 (PE or DVT or VTE).tw. (2560)

8 ((blood flow or vein* or ven*) adj2 stasis).tw. (141)

9 blood clot?.tw. (349)

10 or/1-9 (24224)

11 Immune System Diseases/ (20)

12 Autoimmune Diseases/ (160)

13 Connective Tissue Diseases/ (49)

14 or/11-13 (227)

15 (mixed connective tissue or Raynaud* or Sjogren* or lupus or scleros* or scleroderm* or crest or crst or spondyl?arthrit* or spondyl?arthropath* or psoria* arthrit* or psoria* arthropath* or spondy?lit* or dermatomyosit* or polymyosit*).tw. (8885)

16 14 and 15 (46)

17 Arthritis, Rheumatoid/ (3651)

18 (rheumat* adj2 arthrit*).tw. (6147)

19 Arthritis, Juvenile Rheumatoid/ (178)

20 (juvenile adj4 arthrit*).tw. (311)

21 Caplan Syndrome/ (0)

22 (caplan* adj2 (disease* or syndrome*)).tw. (37)

23 (rheumat* and pneumoconios*).tw. (2)

24 Felty Syndrome/ (0)

25 (Felty* adj2 (disease* or syndrome*)).tw. (37)

26 Rheumatoid Nodule/ (11)

27 (rheumat* adj2 nodul*).tw. (30)

28 Rheumatoid Vasculitis/ (0)

29 (rheumat* adj2 vasculit*).tw. (7)

30 or/17-29 (6715)

31 10 and 30 (179)

32 Mixed Connective Tissue Disease/ (2)

33 (mixed connective tissue adj2 (disorder* or disease* or syndrome*)).tw. (14)

34 (sharp* adj2 (disease* or syndrome*)).tw. (8)

35 overlap disease*.tw. (2)

36 or/32-35 (25)

37 Raynaud Disease/ (264)

38 Raynaud*.tw. (487)

39 or/37-38 (507)

40 Sjogren's Syndrome/ (130)

41 (Sjogren* adj2 (disease* or syndrome*)).tw. (326)

42 (sicca* adj2 syndrome*).tw. (63)

43 or/40-42 (356)

44 Lupus Erythematosus, Systemic/ (346)

45 (system* adj2 lupus).tw. (592)

46 (lupus adj2 (erythematos* or erythromatos* or erithematos* or erythmatos*)).tw. (627)

47 (neonat* adj2 lupus).tw. (3)

48 (libman* adj2 (sach* or sack*)).tw. (3)

49 or/44-48 (702)

50 Lupus Vasculitis, Central Nervous System/ (2)

51 (lupus adj2 vasculit*).tw. (5)

52 (central nervous system adj2 lupus).tw. (4)

53 (CNS adj2 lupus).tw. (2)

54 or/50-53 (12)

55 exp Lupus Erythematosus, Cutaneous/ (18)

56 (cutaneous adj2 lupus).tw. (25)

57 (discoid* adj2 lupus).tw. (25)

58 or/55-57 (43)

59 exp Scleroderma, Systemic/ (281)

60 systemic scleros*.tw. (340)

61 systemic scleroderm*.tw. (18)

62 diffuse scleros*.tw. (5)

63 diffuse scleroderm*.tw. (19)

64 progressive scleros*.tw. (0)

65 progressive scleroderm*.tw. (3)

66 limited scleroderm*.tw. (8)

67 (syndrome* adj2 (crest or crst)).tw. (7)

68 (calcin* adj3 raynaud* adj3 esophag* adj3 sclerod*).tw. (0)

69 or/59-68 (434)

70 Spondylarthritis/ (29)

71 spondyl?arthrit*.tw. (115)

72 (arthrit* adj2 (spine* or spinal)).tw. (19)

73 or/70-72 (135)

74 exp Spondylarthropathies/ (605)

75 spondyl?arthropath*.tw. (80)

76 ((Bechterew* or Bekhterev*) adj2 (disease* or phenomen*)).tw. (24)

77 enteropathic arthrit*.tw. (0)

78 (psoria* adj1 arthrit*).tw. (325)

79 (psoria* adj1 arthropath*).tw. (19)

80 (reactive adj2 (arthrit* or arthropath*)).tw. (68)

81 (reiter* adj2 (disease* or syndrome*)).tw. (36)

82 (post infect* adj2 (arthrit* or arthropath*)).tw. (6)

83 (postinfect* adj2 (arthrit* or arthropath*)).tw. (0)

84 (spondy?lit* adj2 (ankylo* or rheumat*)).tw. (651)

85 or/74-84 (1104)

86 exp Polymyositis/ (32)

87 dermatomyosit*.tw. (77)

88 polymyosit*.tw. (54)

89 multiple myosit*.tw. (0)

90 or/86-89 (96)

91 exp Systemic Vasculitis/ (69)

92 (ANCA adj2 vasculitis).tw. (55)

93 anti neutrophil cytoplasmic antibody associated vasculitis.tw. (7)

94 churg strauss syndrome*.tw. (22)

95 (eosinophilic granulomatosis adj2 polyangiitis).tw. (1)

96 eGPA.tw. (2)

97 wegener* granulomatosis.tw. (83)

98 polyarteritis nodosa.tw. (36)

99 granulomatous vasculitis.tw. (0)

100 Vasculitis, Leukocytoclastic, Cutaneous/ (3)

101 leukocytoclastic vasculitis.tw. (6)

102 small vessel vasculitis.tw. (5)

103 Behcet Syndrome/ (84)

104 Purpura, Schoenlein-Henoch/ (27)

105 Cryoglobulinemia/ (24)

106 behcet syndrome.tw. (12)

107 purpura rheumatica.tw. (0)

108 anaphylactoid purpura.tw. (9)

109 cryoglobulinemia.tw. (27)

110 or/91-109 (341)

111 16 or 30 or 36 or 39 or 43 or 49 or 54 or 58 or 69 or 73 or 85 or 90 or 109 (9337)

112 10 and 111 (285)

********************************************************************

**Embase Final Strategy**

Database: Embase Classic+Embase <1947 to 2014 June 05>

Search Strategy:

--------------------------------------------------------------------------------

1 thrombosis/ (109924)

2 thromboembolism/ (55036)

3 venous thromboembolism/ (19013)

4 exp vein thrombosis/ (95565)

5 lung embolism/ (65220)

6 lung infarction/ (1825)

7 (thrombo* or thrombus* or embol*).tw. (496100)

8 (PE or DVT or VTE).tw. (48120)

9 ((blood flow or vein* or ven*) adj2 stasis).tw. (2884)

10 blood clot?.tw. (5325)

11 or/1-10 (628137)

12 immunopathology/ (18945)

13 autoimmune disease/ (70952)

14 connective tissue disease/ (12457)

15 or/12-14 (99729)

16 (mixed connective tissue or Raynaud* or Sjogren* or lupus or scleros* or scleroderm* or crest or crst or spondyl?arthrit* or spondyl?arthropath* or psoria* arthrit* or psoria* arthropath* or spondy?lit* or dermatomyosit* or polymyosit*).tw. (321561)

17 15 and 16 (19413)

18 rheumatoid arthritis/ (147365)

19 (rheumat* adj2 arthrit*).tw. (115218)

20 juvenile rheumatoid arthritis/ (14810)

21 (juvenile adj4 arthrit*).tw. (10580)

22 pneumoconiosis/ (9361)

23 (caplan* adj2 (disease* or syndrome*)).tw. (237)

24 (rheumat* and pneumoconios*).mp. (388)

25 Felty syndrome/ (990)

26 (Felty* adj2 (disease* or syndrome*)).tw. (913)

27 rheumatoid nodule/ (1590)

28 (rheumat* adj2 nodul*).tw. (1489)

29 rheumatoid vasculitis/ (93)

30 (rheumat* adj2 vasculit*).tw. (704)

31 or/18-30 (183733)

32 11 and 31 (4418)

33 mixed connective tissue disease/ (2344)

34 (mixed connective tissue adj2 (disorder* or disease* or syndrome*)).tw. (2223)

35 (sharp* adj2 (disease* or syndrome*)).tw. (303)

36 overlap disease*.tw. (38)

37 or/33-36 (3318)

38 Raynaud phenomenon/ (11631)

39 Raynaud*.tw. (8886)

40 or/38-39 (13537)

41 Sjoegren syndrome/ (17082)

42 (Sjogren* adj2 (disease* or syndrome*)).tw. (14818)

43 (sicca* adj2 syndrome*).tw. (1001)

44 or/41-43 (20391)

45 systemic lupus erythematosus/ (68384)

46 (system* adj2 lupus).tw. (50376)

47 (lupus adj2 (erythematos* or erythromatos* or erithematos* erythmatos*)).tw. (58540)

48 (neonat* adj2 lupus).tw. (822)

49 (libman* adj2 (sach* or sack*)).tw. (351)

50 or/45-49 (81966)

51 brain vasculitis/ (1829)

52 (lupus adj2 vasculit*).tw. (322)

53 (brain adj2 vasculit*).tw. (78)

54 (central nervous system adj2 lupus).tw. (152)

55 (CNS adj2 lupus).tw. (217)

56 or/51-55 (2486)

57 exp skin lupus erythematosus/ (5261)

58 (cutaneous adj2 lupus).tw. (1906)

59 (discoid* adj2 lupus).tw. (2117)

60 or/57-59 (6168)

61 exp systemic sclerosis/ (20487)

62 systemic scleros*.tw. (14180)

63 systemic scleroderm*.tw. (1517)

64 diffuse scleros*.tw. (747)

65 diffuse scleroderm*.tw. (631)

66 progessive scleros*.tw. (1)

67 progressive scleroderm*.tw. (398)

68 limited scleroderm*.tw. (190)

69 (syndrome* adj2 (crest or crst)).tw. (725)

70 (calcin* adj3 raynaud* adj3 esophag* adj3 sclerod*).tw. (154)

71 or/61-70 (24117)

72 spondylarthritis/ (2249)

73 spondyl?arthrit*.tw. (4035)

74 (arthrit* adj2 (spine* or spinal)).tw. (202)

75 or/72-74 (4761)

76 spondyloarthropathy/ (4007)

77 psoriatic arthritis/ (11434)

78 reactive arthritis/ (2612)

79 ankylosing spondylitis/ (21130)

80 spondyl?arthropath*.tw. (3783)

81 ((Bechterew* or Bekhterev*) adj2 (disease* or phenomenon*)).tw. (747)

82 enteropathic arthrit*.tw. (100)

83 (psoria* adj1 arthrit*).tw. (8518)

84 (reactive adj2 (arthrit* or arthropath*)).tw. (2804)

85 (reiter* adj2 (disease* or syndrome*)).tw. (2880)

86 (post infect* adj2 (arthrit* or arthropath*)).tw. (36)

87 (postinfect* adj2 (arthrit* or arthropath*)).tw. (57)

88 (spondy?lit* adj2 (ankylo* or rheumat*)).tw. (15259)

89 or/76-88 (39779)

90 polymyositis/ (6966)

91 exp dermatomyositis/ (12339)

92 polymyosit*.tw. (6586)

93 dermatomyosit*.tw. (9590)

94 multiple myosit*.tw. (13)

95 or/90-94 (17981)

96 systemic vasculitis/ (2412)

97 ANCA associated vasculitis/ (2268)

98 Churg Strauss syndrome/ (3619)

99 Wegener granulomatosis/ (10075)

100 polyarteritis nodosa/ (8817)

101 rheumatoid vasculitis/ (93)

102 (ANCA adj2 vasculitis).tw. (1905)

103 anti neutrophil cytoplasmic antibody associated vasculitis.tw. (76)

104 churg strauss syndrome*.tw. (2269)

105 (eosinophilic granulomatosis adj2 polyangiitis).tw. (105)

106 eGPA.tw. (81)

107 wegener* granulomatosis.tw. (6947)

108 polyarteritis nodosa.tw. (3406)

109 granulomatous vasculitis.tw. (573)

110 Behcet disease/ (12081)

111 anaphylactoid purpura/ (5780)

112 cryoglobulinemia/ (5089)

113 behcet syndrome.tw. (277)

114 purpura rheumatica.tw. (66)

115 anaphylactoid purpura.tw. (456)

116 cryoglobulinemia.tw. (3466)

117 or/96-116 (46444)

118 or/17,31,37,40,44,50,56,60,71,75,89,95,117 (376228)

119 11 and 118 (17279)

120 exp cohort analysis/ (169019)

121 exp longitudinal study/ (67650)

122 exp prospective study/ (252972)

123 exp follow up/ (826350)

124 cohort$.tw. (398717)

125 exp case control study/ (85414)

126 (case$ and control$).tw. (465108)

127 exp case study/ (35379)

128 (case$ and series).tw. (190062)

129 or/120-128 (1950462)

130 119 and 129 (2960)

********************************************************************

**Medline Final Strategy**

Database: Ovid MEDLINE(R) In-Process & Other Non-Indexed Citations, Ovid MEDLINE(R) Daily and Ovid MEDLINE(R) <1946 to Present>

Search Strategy:

--------------------------------------------------------------------------------

1 Thrombosis/ (55032)

2 Thromboembolism/ (20324)

3 Venous Thromboembolism/ (4562)

4 exp Venous Thrombosis/ (44424)

5 exp Pulmonary Embolism/ (31272)

6 (thrombo* or thrombus* or embol*).tw. (349117)

7 (PE or DVT or VTE).tw. (32379)

8 ((blood flow or vein* or ven*) adj2 stasis).tw. (1755)

9 blood clot?.tw. (3632)

10 or/1-9 (412792)

11 Immune System Diseases/ (3757)

12 Autoimmune Diseases/ (43001)

13 Connective Tissue Diseases/ (4472)

14 or/11-13 (50651)

15 (mixed connective tissue or Raynaud* or Sjogren* or lupus or scleros* or scleroderm* or crest or crst or spondyl?arthrit* or spondyl?arthropath* or psoria* arthrit* or psoria* arthropath* or spondy?lit* or dermatomyosit* or polymyosit*).tw. (230050)

16 14 and 15 (8279)

17 Arthritis, Rheumatoid/ (81514)

18 (rheumat* adj2 arthrit*).tw. (79075)

19 Arthritis, Juvenile Rheumatoid/ (8337)

20 (juvenile adj4 arthrit*).tw. (7486)

21 Caplan Syndrome/ (141)

22 (caplan* adj2 (disease* or syndrome*)).tw. (114)

23 (rheumat* and pneumoconios*).tw. (73)

24 Felty Syndrome/ (623)

25 (Felty* adj2 (disease* or syndrome*)).tw. (682)

26 Rheumatoid Nodule/ (849)

27 (rheumat* adj2 nodul*).tw. (955)

28 Rheumatoid Vasculitis/ (17)

29 (rheumat* adj2 vasculit*).tw. (494)

30 or/17-29 (112044)

31 10 and 30 (1753)

32 Mixed Connective Tissue Disease/ (1471)

33 (mixed connective tissue adj2 (disorder* or disease* or syndrome*)).tw. (1740)

34 (sharp* adj2 (disease* or syndrome*)).tw. (208)

35 overlap disease*.tw. (21)

36 or/32-35 (2316)

37 Raynaud Disease/ (5805)

38 Raynaud*.tw. (5875)

39 or/37-38 (8006)

40 Sjogren's Syndrome/ (10250)

41 (Sjogren* adj2 (disease* or syndrome*)).tw. (11633)

42 (sicca* adj2 syndrome*).tw. (760)

43 or/40-42 (13912)

44 Lupus Erythematosus, Systemic/ (45262)

45 (system* adj2 lupus).tw. (38538)

46 (lupus adj2 (erythematos* or erythromatos* or erithematos* or erythmatos*)).tw. (44347)

47 (neonat* adj2 lupus).tw. (637)

48 (libman* adj2 (sach* or sack*)).tw. (227)

49 or/44-48 (58438)

50 Lupus Vasculitis, Central Nervous System/ (549)

51 (lupus adj2 vasculit*).tw. (223)

52 (central nervous system adj2 lupus).tw. (137)

53 (CNS adj2 lupus).tw. (157)

54 or/50-53 (952)

55 exp Lupus Erythematosus, Cutaneous/ (4011)

56 (cutaneous adj2 lupus).tw. (1420)

57 (discoid* adj2 lupus).tw. (1382)

58 or/55-57 (4743)

59 exp Scleroderma, Systemic/ (16562)

60 systemic scleros*.tw. (9977)

61 systemic scleroderm*.tw. (1083)

62 diffuse scleros*.tw. (413)

63 diffuse scleroderm*.tw. (407)

64 progressive scleros*.tw. (117)

65 progressive scleroderm*.tw. (232)

66 limited scleroderm*.tw. (139)

67 (syndrome* adj2 (crest or crst)).tw. (572)

68 (calcin* adj3 raynaud* adj3 esophag* adj3 sclerod*).tw. (140)

69 or/59-68 (19414)

70 Spondylarthritis/ (847)

71 spondyl?arthrit*.tw. (2261)

72 (arthrit* adj2 (spine* or spinal)).tw. (125)

73 or/70-72 (2587)

74 exp Spondylarthropathies/ (18317)

75 spondyl?arthropath*.tw. (2892)

76 ((Bechterew* or Bekhterev*) adj2 (disease* or phenomen*)).tw. (417)

77 enteropathic arthrit*.tw. (58)

78 (psoria* adj1 arthrit*).tw. (5148)

79 (psoria* adj1 arthropath*).tw. (469)

80 (reactive adj2 (arthrit* or arthropath*)).tw. (2286)

81 (reiter* adj2 (disease* or syndrome*)).tw. (1939)

82 (post infect* adj2 (arthrit* or arthropath*)).tw. (31)

83 (postinfect* adj2 (arthrit* or arthropath*)).tw. (44)

84 (spondy?lit* adj2 (ankylo* or rheumat*)).tw. (9763)

85 or/74-84 (24538)

86 exp Polymyositis/ (7463)

87 dermatomyosit*.tw. (6377)

88 polymyosit*.tw. (4778)

89 multiple myosit*.tw. (12)

90 or/86-89 (10901)

91 exp Systemic Vasculitis/ (12955)

92 (ANCA adj2 vasculitis).tw. (1302)

93 anti neutrophil cytoplasmic antibody associated vasculitis.tw. (68)

94 churg strauss syndrome*.tw. (1709)

95 (eosinophilic granulomatosis adj2 polyangiitis).tw. (66)

96 eGPA.tw. (46)

97 wegener* granulomatosis.tw. (5424)

98 polyarteritis nodosa.tw. (2478)

99 granulomatous vasculitis.tw. (438)

100 Vasculitis, Leukocytoclastic, Cutaneous/ (1683)

101 leukocytoclastic vasculitis.tw. (1177)

102 small vessel vasculitis.tw. (806)

103 Behcet Syndrome/ (7333)

104 Purpura, Schoenlein-Henoch/ (3504)

105 Cryoglobulinemia/ (2552)

106 behcet syndrome.tw. (188)

107 purpura rheumatica.tw. (24)

108 anaphylactoid purpura.tw. (246)

109 cryoglobulinemia.tw. (2671)

110 or/91-109 (31929)

111 16 or 30 or 36 or 39 or 43 or 49 or 54 or 58 or 69 or 73 or 85 or 90 or 110 (250429)

112 10 and 111 (9313)

113 exp cohort studies/ (1355031)

114 cohort$.tw. (280601)

115 controlled clinical trial.pt. (88505)

116 epidemiologic methods/ (29795)

117 exp case-control studies/ (663685)

118 (case$ and control$).tw. (331545)

119 (case$ and series).tw. (122933)

120 or/113-119 (1983045)

121 112 and 120 (1987)

122 remove duplicates from 121 (1961)

********************************************************************
